# Effects of Hearing Disability on the Employment Status Using WHODAS 2.0 in Taiwan

**DOI:** 10.3390/ijerph17249374

**Published:** 2020-12-15

**Authors:** Pin-Zhir Chao, Shih-Wei Huang, Reuben Escorpizo, Wen-Chou Chi, Chia-Feng Yen, Hua-Fang Liao, Yi-Wen Chen, Tsan-Hon Liou

**Affiliations:** 1 Department of Otolaryngology, Shuang Ho Hospital, Taipei Medical University, New Taipei 23561, Taiwan; 09312@s.tmu.edu.tw; 2 Department of Otolaryngology, School of Medicine, College of Medicine, Taipei Medical University, Taipei 11031, Taiwan; 3 Department of Physical Medicine and Rehabilitation, Shuang Ho Hospital, Taipei Medical University, New Taipei 23561, Taiwan; 13001@s.tmu.edu.tw (S.-W.H.); 17304@s.tmu.edu.tw (Y.-W.C.); 4 Department of Physical Medicine and Rehabilitation, School of Medicine, College of Medicine, Taipei Medical University, Taipei 11031, Taiwan; 5 Department of Rehabilitation and Movement Science, College of Nursing and Health Sciences, University of Vermont, Burlington, VT 05405, USA; escorpizo.reuben@gmail.com; 6 Swiss Paraplegic Research, 6207 Nottwil, Switzerland; 7 Taiwan Society of International Classification of Functioning, Disability and Health, TSICF, New Taipei 23561, Taiwan; y6312002@gmail.com (W.-C.C.); mapleyeng@mail.tcu.edu.tw (C.-F.Y.); hfliao@ntu.edu.tw (H.-F.L.); 8 Department of Occupational Therapy, College of Medical Sciences and Technology, Chung Shan Medical University, Taichung 40201, Taiwan; 9 Department of Public Health, Tzu Chi University, Hualien 97004, Taiwan; 10 School and Graduate Institute of Physical Therapy, College of Medicine, National Taiwan University, Taipei 10617, Taiwan

**Keywords:** employment, working status, WHODAS 2.0, ICF, hearing loss, hearing disability

## Abstract

The aim of this study was to explore the association between employment status and World Health Organization Disability Assessment Schedule, Second Edition (WHODAS 2.0) scores of working-age subjects with hearing impairment. The data of 18,573 working-age subjects (age ≥ 18 and <65 years) with disabling hearing impairment were obtained from the Taiwan Data Bank of Persons with Disability (TDPD) for the period from 11 July 2012 to 31 October 2018. Demographic data and WHODAS 2.0 scores for each domain were analyzed to identify their relationship with employment status. Unemployed subjects with disabling hearing impairment had higher WHODAS 2.0 scores in all domains compared with the employed subjects. Binary logistic regression revealed that older age, female sex, lower educational level, institutional residence, rural residence, lower family income, and moderate to severe impairment were more strongly associated with unemployment status. The data in this large population-based study offer comprehensive information on important factors associated with the employment status of people with disabling hearing impairment. Early identification of risks of unemployment of patients with hearing impairment can raise awareness for aggressive community and government campaigns regarding public health to improve the self-confidence, social participation, and related psycho-social wellbeing of people.

## 1. Introduction

According to a World Health Organization (WHO) report updated in March 2018, more than 5% of the world population (estimated 466 million people) had disabling hearing loss compared with 360 million people in 2013. Moreover, it is estimated that more than 900 million people will have disabling hearing loss by 2050, which is approximately one in every 10 people [1]. Disabling hearing loss was defined as an average loss of 30 dB or worse across frequencies in the better-hearing ear. Hearing loss is among the top five causes of years lived with disability (YLDs), which is a metric for assessing the impact of a disabled health condition globally [2]. The total number of people with disabling hearing loss is expected to increase due to greater exposure to damagingly loud sound, prolonged life expectancy globally, and a rapid population growth in developing countries where hearing loss risk is higher than that in developed countries. The prevalence and number of YLDs due to hearing loss was substantially higher than before in a recent global burden of disease study [3]. The study results indicated that hearing loss is an immense burden and a global health concern. In Taiwan, the number of hearing-impaired people reached 122,988 in 2014, accounting for 10.71% of the total disabled population of Taiwan. It is also the third largest group of disability category in Taiwan [4]. The WHO estimated that unaddressed hearing loss poses an annual global cost of 750 billion international dollars, including loss of educational support and productivity, accompanying a much higher unemployment rate and underemployment conditions in people with hearing loss [5].

To prevent a disability becoming a handicap, a framework was developed by the WHO to evaluate the functions and disability conditions in a holistic and multidimensional manner as the International Classification of Functioning, Disability and Health (ICF) in 2001. The ICF links bodily function and structure with activities limitations and participation restrictions by considering environmental and personal factors and incorporating them into a bio-psycho-social model composed of “Health Condition,” “Contextual Factors,” “Body Functions and Structures,” and “Activity and Participation” for understanding the roles and significance of impairments in the view of individuals and society as a whole. It was designed as an international standard and a comparative benchmark for describing and measuring the effects of health conditions on the functionality and disability worldwide [6]. ICF core sets had been developed and associated with 34 specific disorders or disease states until 2015 [7], including in a plethora of studies published in the hearing impairment category [8,9,10,11].

To comprehensively evaluate and quantify the disability level, an objective assessment tool that measures physical and psycho-social aspects is essential. On the basis of the ICF framework concepts, the WHO Disability Assessment Schedule, Second Edition (WHODAS 2.0) was proposed by the WHO as a feasible tool for assessing factors associated with functional disability and analyzing the risk factors responsible for these disabilities, while closely corresponding to the ICF component of activities and participation [12]. WHODAS 2.0 uses 36 items for assessing the following six domains: cognition, mobility, self-care, getting along with people, life activities, and participation. Domain scores aggregate to a total score, with a higher score implicating a more severely disabled condition, providing a summary of functioning and disabilities that is reliable and applicable. The psychometric properties of WHODAS 2.0 has been evaluated for numerous clinical conditions, including osteoarthritis [13], stroke [14], psychiatric disorders [15], and cancer [16]. It has also been validated and consolidated in several studies and has been applied for evaluating the disability status of hearing disability [17]. It has been translated into 47 languages and used in 27 areas of research with hundreds of studies published till the end of 2017 [18].

Cornelius et al. used WHODAS 2.0 to analyze the association between the six subdomains of disabled individuals and future work status, but only focused on the possibility of working retrieval in relation to unemployment benefit claim [19]. Our previous study confirmed that WHODAS 2.0 is a feasible tool for assessing functional disability in visual impairment [20]. The characteristic employment outcome for people with schizophrenia [21], victims of stroke [22], and survivors of head and neck cancer was proposed using WHODAS 2.0 [23].

However, the data for the effect of hearing disability on working status are lacking. An association of hearing loss with decreased employment and less wage income (odd ratios, 2.2–2.5, *p* < 0.001) among adults in the United States was proposed based on the 2006 and 2008 Medical Expenditure Panel Survey; however, other advanced psycho-social factors were not investigated [24]. Studies on social models of impacts of hearing impairment in activities of daily life were evaluated mostly by self-reported, quality of life measurement, like the SF-36 or Index of Social Interaction (ISI), which may carry an inherent disadvantage of reliability and conflict-of-interest issues and pose a serious challenge to validity. The concept of “Deaf community” and “Identity with Deaf Culture” advocated by Harlan Lane and other psychiatrists referring psychological stress as adjustment difficulties further enhance the phenomenon that deaf community do not see themselves as disabled, but the reluctance of society to provide accommodation and other means of access to communications what they see as profoundly disabling [25]. This makes a multifaceted evaluation of the factors contributing to the unemployment of Deaf and Hard-of-Hearing persons more emergent. This large-scale, population-based study analyzed the related disability factors influencing the working outcomes in Taiwanese people with hearing impairment by using the WHODAS 2.0 assessment tool in the ICF framework. We hypothesized that the WHODAS 2.0 tool can objectively assess the prediction of the employment status of working-age people with hearing impairment to facilitate clinicians to formulate an effective rehabilitation plan and promote efficient public health policies for these individuals.

## 2. Materials and Methods

In 2012, a new disability evaluation process, Disability Eligibility Determination Scale (DES-2012), was developed on the basis of the ICF framework for individuals with functional impairment to apply for disability certification and receive social welfare subsidy from the government in Taiwan. Simultaneously, the Taiwan Data Bank of Persons with Disability (TDPD) was established [26]. The DES-2012 comprised two stages of assessment, and each is performed in person. In the first stage, the body function (b codes) and body structure (s codes) categories of the ICF were assessed with DES-2012 coding criteria by a clinical physician specialized in the field. The physician then assigned a diagnostic code to the disease in accordance with the International Classification of Diseases, Ninth Revisions, Clinical Modification (ICD-9-CM) and ICD-10-CM codes. In the second stage, the environmental categories (e codes) of the ICF framework were assessed by a trained specialist, such as a physical therapist, occupational therapist, speech pathologist, psychologist, or social worker. In addition, the specialist evaluated social participation status and restriction in life activities of the patient by using WHODAS 2.0 (traditional Chinese version) [27]. After the DES-2012 process was completed, the data of each disabled patient were registered in the TDPD database and were de-identified for privacy protection.

**Figure 1:**
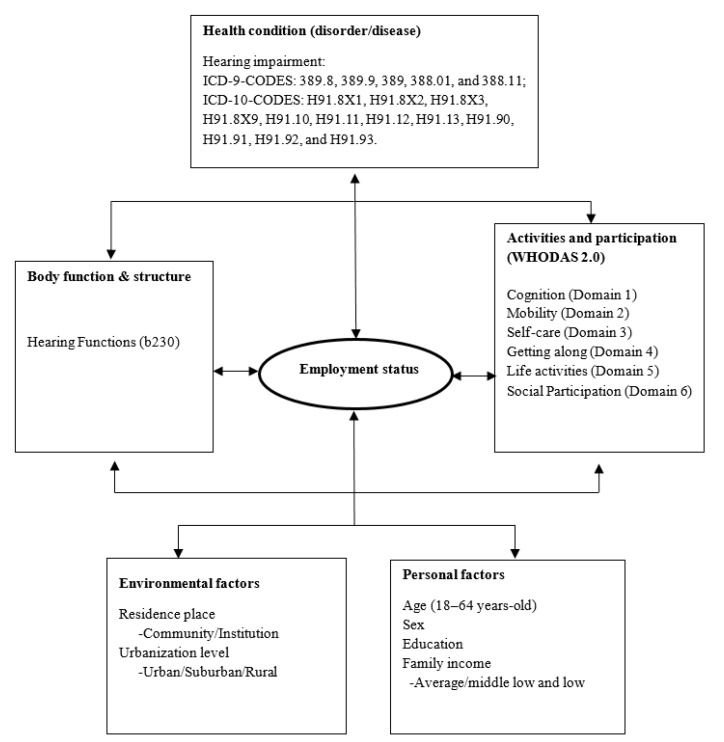
International Classification of Functioning, Disability and Health (ICF) framework of employment status among people with hearing impairment.

**Figure 2:**
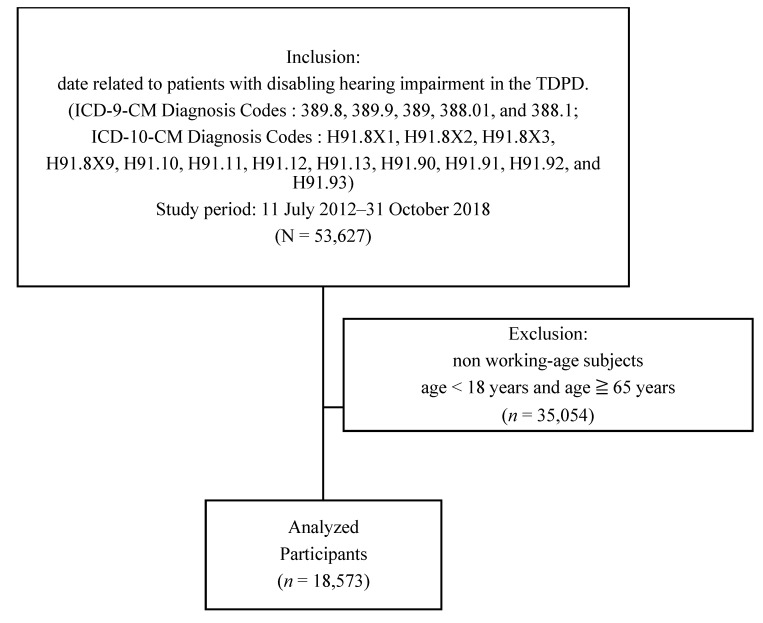
Sample selection flowchart.

A total of 18,573 patients with hearing disability aged between 18 and 64 years from 11 July 2012, to 31 October 2018, were enrolled from the TDPD database. The enrolled patients had ICD-10-CM codes H91.8X1, H91.8X2, H91.8X3, H91.8X9, H91.10, H91.11, H91.12, H91.13, H91.90, H91.91, H91.92, and H91.93 or ICD-9-CM codes 389.8, 389.9, 389, 388.01, and 388.11. Demographic data such as age, sex, residential status (community dwelling or institution), urbanization level (rural, suburban, or urban), socioeconomic status of family (above average, middle, or low), education level (above college, senior high school, junior high school, or under junior high school), employment status, and WHODAS 2.0 scores (traditional Chinese version) were obtained from TDPD. ICF categories with severity of body function and body structure were recorded, and results revealed severe hearing impairment (ICF category b230). Mild, moderate, and severe disability in hearing function were defined as hearing handicap (HH) in both ears between 50% and 70%, between 71% and 90%, and >90%, respectively, with b230.3 implicating severe impairment. Patients younger than 18 years or older than 65 years were excluded from the study because they were not within the legal age of labor force of Taiwan (Figure 1 and Figure 2).

The employment status was defined as working either full time or part time with salary level meeting the regulation set by the Department of Labor in Taiwan. The definition of the severity of hearing impairment indicated as HH with percentage loss of hearing worksheet, suggested and modified by American Academy of Otolaryngology by using pure-tone average of thresholds at frequencies 0.5, 1, 2, and 4 KHz in calculating equations [28]. This study was approved by the Joint Institutional Review Board at Taipei Medical University (Approval No. N201805048) and informed consent was waived because this was a retrospective secondary data analysis.

### 2.1. Outcome Measurements

The 36-item traditional Chinese version of WHODAS 2.0 scores was designed by authorized specialists after they interviewed the patients (or their proxies if patients were not able to answer the questions). WHODAS 2.0 has six domains and 36 items in total, including six items on cognition (Domain 1); five items on mobility (Domain 2); four items on self-care (Domain 3); five items on getting along with people (Domain 4); eight items on life activities (4 items for household activities and 4 items for work or school activities; Domain 5), and eight items on social participation (Domain 6). The patients indicated their level of difficulty in performing activities related to each item in the past 30 days on a 5-point Likert scale (1 = no difficulty; 2 = mild difficulty; 3 = moderate difficulty; 4 = severe difficulty; and 5 = extreme difficulty). Each domain score and summary scores were transformed to standardized scores ranging from 0 to 100, with higher scores indicating higher severity of disability [27]. The four items in Domain 5 regarding work or school activities were excluded because our study investigated the predictors of employment status in patients with hearing impairment. The traditional Chinese version of WHODAS 2.0 was validated for interclass correlation, internal consistency, and reliability [29]. The WHODAS 2.0 manual allows up to 30% of the items in each domain to be unrated for score computation; the missing values can be adjusted using the mean of the available scores in that domain [12].

### 2.2. Statistical Analysis

The demographic data are presented as numbers and percentages. The chi-square test was used to compare categorical variables between male and female patients. Patients were classified into two groups, namely employed and unemployed, depending on their work status when undergoing the DES-2012 assessment process. The Wilcoxon rank sum test was used for comparing the continuous variables of the standardized WHODAS 2.0 summary and six domain scores between the employed and unemployed groups. We applied binary logistic regression to determine the unemployment risk by using baseline variables, such as age, sex, education, residence, urbanization level, family income, and severity of impairment. The data were analyzed using SAS 9.4 (SAS Institute, Inc., Cary, NC, USA). The differences or correlations were considered significant if the *p* value was < 0.05.

## 3. Results

Data of 53,627 subjects with disabling hearing impairment were obtained from the TDPD database. In this study, data of working-age subjects (age ≥ 18 and <65 years) were selected, and 18,573 subjects were identified and analyzed (men, *n* = 10,155 and women, *n* = 8418).

The demographic characteristics and employment status of the participants are presented in Table 1. The domain-specific and summary scores of WHODAS 2.0 were higher in the unemployment group than in the employment group, indicating that the unemployment group had a higher disability status (*p* < 0.001) (Table 2).

To quantify the domain scores, we determined the scores below 5% of the total scales as the extent of negative effect according to ICF qualifier [30] (p. 22).

Quantified WHODAS 2.0 scores and variables were analyzed using binary logistic regression analysis to determine risk factors for unemployment (Table 3). The results revealed that age, sex, education level, residence, urbanization level, family income, and severity of impairment were associated with the employment status.

## 4. Discussion

This study examined the contributing factors of employment conditions and the relationship between disability and employment status by using WHODAS 2.0 in a large group of individuals with hearing impairment in Taiwan. As previous researchers argued by describing the association of the severity of disability and employment [31], our study indicated that hearing disabled participants with more significant functional impairment as measured by WHODAS 2.0 exhibited a higher rate of unemployment.

Individuals with moderate to severe hearing loss were found more likely to have impaired activities of daily living than those with mild hearing loss or normal hearing ability [32]. In addition to the direct effects of sensory dysfunction, poor communication, and cognitive ability, the indirect effects of hearing loss included functional, psycho-social, and economic aspects. Reasons for indirect impacts included limited services and exclusion from society, resulting in loneliness, isolation, frustration, marginalization, stigmatization, and safety concerns arising from not being able to hear a car horn or other warning sounds, particularly among elderly people [33]. For people of working age, the difficulties in managing certain job situations include being unable to meet the demands of competitive employment conditions and decreasing social problem-solving skills, leading to a negative influence on their relationships with family and a low motivation level for social participation, which is further a key barrier to returning to work [34,35]. For people with hearing impairment, obtaining employment or returning to work was thought to strengthen their self-empowerment and the feeling of accomplishment, provide financial remuneration and self-determination, and enhance social participation because vocation is viewed as a key component of overall functioning [21].

Our study was unique in that the disability severity was quantified objectively using WHODAS 2.0, an assessment tool for evaluating the functional impairment of participants in the area of cognition, mobility, self-care, getting along, life activity, and social participation, in addition to perception, because several previous studies have solely focused on the influence of physical factors of impairment and have suggested the correlation between hearing disability and employment outcome. A corresponding assessment for accurately predicting the employment status must be aware of the importance of a holistic range of both symptoms and functional outcomes and comprise multidimensional instruments to make the comparison between studies more feasible, as ICF-based WHODAS 2.0 did [24]. Such a concept has not been fully utilized in the audiology literature for elucidating the employment status of the patients with hearing disability [18].

A lower disability status in all the WHODAS 2.0 subdomains was observed in patients with hearing disability having a working status compared with those without. Notably, the domains of mobility, self-care, and life activities pertain to basic activities of daily life and are therefore partially related to the employment status, whereas the functional aspects of understanding and communicating, getting along with people, and social participation require more advanced skills and are necessary for maintaining a job.

According to the ICF framework, a person’s functionality or disability represents a dynamic interaction between health conditions and contextual factors, such as environmental and personal factors. The daily activity is affected by a disability in the health condition, which then causes a decline in cognitive functions, self-care, and social participation, forming a vicious cycle [17]. Social participation offers and reinforces social roles, for instance, joining community activities and maintaining friendship can provide a sense of social belonging, widen social network, and deepen self-esteem in daily life. Environmental factors, such as the state of domestic or global economy, social welfare policies, and barrier-free space provision, also play roles in social participation. The comprehensiveness of assessment can be achieved by application of the WHODAS 2.0 tool [36].

The logistic regression analysis in our study revealed that multiple variables, including age, sex, socioeconomic status, urbanization level, residence, education, and severity of impairment, were associated with employment status. This finding was partly supported by a study on the socioeconomic impact of hearing loss in adults in the United States from 1999 to 2002 by the National Health and Nutritional Examination Survey, which revealed that hearing loss was associated with low educational attainment, low income, and unemployment or underemployment [37]. WHO estimates of global burden of disabling hearing impairment have increased substantially. The cost of communication disorders by considering rehabilitation, special education, and loss of employment amounted to 2.5–3% of general national products in the USA in 1999 [38]. In addition to reduced productivity, people with hearing loss may avoid a demanding and threatening situation out of fear of embarrassment, frustration, isolation, and stress and where they have little control over the environments, such as at the workplace or around casual acquaintance with strangers [39]. Further, people with hearing disabilities have been demonstrated to be less likely to have paid jobs than normal hearing individuals, especially women and those with a low educational level, and are more vulnerable to the impacts of economic downturns [40,41]. From the internal perspective, people with hearing impairment often feel being isolated from society and stigmatized and hence tend to avoid being labeled as hearing disabled, using hearing aids, and taking hearing tactics. A threshold effect associated with the relationship between hearing loss and poor health condition was proposed with health-related effects rising drastically at and beyond moderate levels of hearing disability [42].

Sex may also influence the effect of hearing loss on socialization. Evidence suggested that men may attribute a lower value to communication than women and may not search for solutions actively. They may more likely withdraw from social activities than struggle to maintain participation. In our study, female subjects with hearing disability were two times less likely to be employed compared with male subjects with hearing disability. By contrast, men with hearing impairment imposed their needs more strongly on their family than women; therefore, women reported a feeling of being less understood by their spouses than men [43].

We found that low education level significantly discriminated employment conditions. According to recent data from East Asian countries, the unemployment rate of highly educated people was above average, and people with low educational level had the highest unemployment rate. These facts may be related to the supply–demand mismatch of the current labor market and the high education system in this area [44]. Furthermore, the national health insurance and low-income subsidy welfare in Taiwan have reduced the medical and living expense burden of individuals with hearing disability for them to retire earlier rather than returning to work, resulting in discrepancies observed in the analysis of correlation of educational level and socioeconomic status with employment [45].

In this study, the WHODAS 2.0 assessment tool was introduced as a measurement tool for hearing disability. Previous hearing loss-related test tools or questionnaires have caused the problem that the segmental aspects related to hearing loss do not sufficiently explain the correlation between health and environmental factors and functional aspects. Nevertheless, WHODAS 2.0 may not be sufficiently sensitive to detect the quality of life change in hearing-related disability. Ho et al. reported no significant correlation between Hearing Handicap Inventory for Adults or Hearing Handicap Inventory for Elderly Screening and any WHODAS score (all *p* > 0.05) [46].

Considering the theoretical framework of the ICF, there seems to be a gap between the actual needs of individuals with hearing loss and the formal support provided by existing public health policies of the government. Therefore, multiple factors need to be addressed when rehabilitation-delivered vocational services and interventional plans for individuals are initiated. Community intervention with social participation should be implemented to maintain a better social interaction ability for social and psychological wellbeing. Running educational campaigns will result in higher rates of employee participation and substantive cost savings, improved rehabilitative service usage, and increased awareness, especially among employers. Employees with hearing impairment should be aware that access to information is imperative to secure workplace adjustments to meet their needs. The barriers towards developing less strenuous working conditions for employees with hearing impairment needs to be recognized and treated accordingly [47]. Early identification of risk factors that caused learned helplessness or fatigue may help vocational rehabilitation professionals supporting employees with hearing impairment and their employers in making adjustments at the workplace and providing vocational training. This can be achieved through legislation support at the national or global level for protection of the rights of people with hearing disability, promoting development of innovative skills and tools to achieve efficient and successful functional rehabilitation [48].

Our study has the following limitations. The employment statuses were categorized on the basis of whether the subject had a regular job or was jobless. There might be circumstances where some were part-time workers or quit their jobs willingly for taking care of disabled elders or for raising a child at home, the so-called “hidden unemployed”. Future studies can further explore stratification of the job types more specifically to determine the effect of disability severity in patients with hearing loss on their respective jobs. WHODAS assessment was performed on the basis of responses given by subjects or their caregivers. Because some people with severe hearing impairment had limited ability to communicate with the interviewers, their assessment was completed by proxies or using communication card, thereby posing the risk of underestimation of the functional disability. In addition, the WHODAS 2.0 questionnaire only evaluated the disability condition of individuals in the past 30 days and was a single-time-point because we were unable to follow up the return to work processes or detect the ability to maintain a stable vocational status of the patient. Thus, the etiology of hearing impairment could not be identified, and we were unable to discriminate some other possible associated factors, such as hearing loss during current career stage or hearing loss during years in education. Because the DES-2012 evaluation has been used to determine the subsidy for people with disabilities in Taiwan, some participants may purposely make false reports regarding their actual capabilities or performance to receive a greater subsidy. Because WHODAS did not consider some environmental factors, such as the impact of global economic turmoil, social welfare, and insurance policies imposed in different countries or family support and marital status, our study results may be limited to a single country.

In all, strategies to mitigate the effect of hearing loss on job security and recruitment must be developed by applying vocational counseling and social education. Effective population-based preventive interventions should be beyond clinical service and traditional areas of diagnosis or treatment and hearing assistive device application. It must focus more on epidemiological surveillance and health promotion to help identify the needs of population, to measure cost and benefits of prevention, to raise awareness regarding the size of problem, to determine program priorities, and to select targets and strategies for prevention. Early identification of problems can be crucial in limiting the level of hearing impairment disabilities with implication for the social security system, leading to effective rehabilitation strategies and policies implementing prioritization of resource allocation.

## 5. Conclusions

This is the first study to combine physical, social and psychometrical factors in the framework of WHODAS 2.0 that tried to encompass the multi-faceted influence of elements for the impact of hearing impairment on employment. The data in this large population-based study offer comprehensive information on important factors associated with the employment status in people with disabling hearing impairment. The unemployment group had a higher domain-specific and summary scores of WHODAS 2.0. Older age, female sex, lower educational level, institutional residence, rural residence, lower family income, and moderate to severe impairment were associated with the unemployment status. Early identification of hearing impairment people at risk of unemployment can raise awareness of public health for aggressive community and government campaigns to improve their self-confidence, social participation and related psycho-social wellbeing.

## Figures and Tables

**Table 1:** Association between sex and sociodemographic characteristics of patients with hearing impairment in Taiwan (*n* = 18,573).

Variables	Men *n* = 10,155		Women *n* = 8418		*p* Value
	No.	%	No.	%	
Age (years)					<0.001
Mean, SD	53.76	10.834	52.96	10.990	
Education					<0.001
≥College	497	4.89	263	3.12	
Senior High	1782	17.55	1260	14.97	
Junior High	4977	49.01	3926	46.64	
≤Primary	2899	28.55	2969	35.27	
Residence					<0.001
Community dwelling	10,094	99.40	8400	99.79	
Institution	61	0.60	18	0.21	
Urbanization level					<0.001
Rural	1501	14.78	1065	12.65	
Suburban	4271	42.06	3541	42.06	
Urban	4383	43.16	3812	45.28	
Family income					0.1352
Average	10,057	99.03	8354	99.24	
Middle low and low	98	0.97	64	0.76	
Severity of Hearing functions					0.0830
Mild	6351	62.54	5316	63.15	
Moderate	2574	25.35	2026	24.07	
Severe	1230	12.11	1076	12.78	
Work Status					<0.001
Employed	4864	47.90	2570	30.53	
Unemployed	5291	52.10	5848	69.47	

(1) Employed includes employment in a firm and own business; (2) Unemployed includes volunteering, student, housekeeper, retired, no work cause health issue or not, and other issue. (3) *p* < 0.05 (4) Wilcoxon rank sum test: age (years). (5) Chi-square test: education, residence, urbanization level, family income, severity of hearing loss, and work status.

**Table 2:** Comparison of employment status with overall disability as well as each domain on Table 2. 0 scores in subjects with hearing impairment in Taiwan (*n* = 18,573).

Variable	Employment (*n* = 7434)		Unemployment (*n* = 11,139)		*p* Value
	Mean	SD	Mean	SD	
Cognition	18.15	16.001	25.05	20.568	<0.001
Mobility	4.91	11.311	12.44	19.353	<0.001
Self-care	2.04	7.054	5.40	12.738	<0.001
Getting along	30.85	24.991	39.24	28.194	<0.001
Life activities	9.18	18.245	19.03	27.560	<0.001
Social Participation	24.10	18.933	30.22	21.768	<0.001
Summary	16.33	12.315	23.27	16.898	<0.001

(1) Testing by Wilcoxon rank sum test.

**Table 3:** Binary logistic regression of WHODAS 2.0 scores for work status, degree of impairment, and basic characteristics.

	Univariate Model					Multivariate Model				
Variables	Beta (β)	OR	99% CI		*p* Value	Beta (β)	OR (Adjusted)	99% CI		*p* Value
Age (year)	0.044	1.045	1.042	1.048	<0.001	0.037	1.038	1.035	1.041	<0.001
Sex (ref. = Male)										
Female	0.738	2.092	1.969	2.222	<0.001	0.796	2.216	2.079	2.363	<0.001
Education (ref. ≥ College)										
Senior High	0.301	1.351	1.151	1.586	<0.001	0.185	1.203	1.019	1.420	0.0288
Junior high	0.467	1.596	1.374	1.853	<0.001	0.286	1.331	1.140	1.554	<0.001
≤Primary	1.451	4.267	3.651	4.986	<0.001	0.948	2.579	2.189	3.039	<0.001
Residence (ref. = Community dwelling)										
Institution	1.781	5.936	2.859	12.325	<0.001	1.966	7.140	3.368	15.138	<0.001
Urbanization level (ref. = urban)										
Suburban	0.072	1.074	1.009	1.144	0.0260	0.061	1.253	1.137	1.382	0.0746
Rural	0.224	1.252	1.142	1.372	<0.001	0.226	1.063	0.994	1.137	<0.001
Family Income (ref. = Average)										
Middle Low and Low	1.338	0.291	0.964	1.857	0.0818	0.376	1.457	1.024	2.072	0.0364
Severity (ref. = Mild)										
Moderate	0.156	1.169	1.090	1.254	<0.001	0.154	1.167	1.083	1.257	<0.001
Severe	0.308	1.360	1.239	1.493	<0.001	0.424	1.528	1.384	1.688	<0.001
Domain Score										
Cognition	0.358	1.431	1.319	1.553	<0.001					
Mobility	0.917	2.501	2.346	2.666	<0.001					
Self-care	0.834	2.304	2.122	2.501	<0.001					
Getting along	0.338	1.401	1.305	1.505	<0.001					
Life activities	0.735	2.085	1.957	2.221	<0.001					
Social Participation	0.406	1.500	1.390	1.620	<0.001					

(1) *p* < 0.05; (2) Domain score cut by < 5 score by ICF manual rule. (WHODAS Score ≥ 5 then Domain Score = 1, else Domain Score = 0); (3) Work status: employment = 0, unemployment = 1; (4) ref., reference.
